# Thin-Film Luminescent Solar Concentrator Based on Intramolecular Charge Transfer Fluorophore and Effect of Polymer Matrix on Device Efficiency

**DOI:** 10.3390/polym13213770

**Published:** 2021-10-31

**Authors:** Fahad Mateen, Namcheol Lee, Sae Youn Lee, Syed Taj Ud Din, Woochul Yang, Asif Shahzad, Ashok Kumar Kaliamurthy, Jae-Joon Lee, Sung-Kyu Hong

**Affiliations:** 1 Department of Chemical and Biochemical Engineering, Dongguk University, Seoul 04620, Korea; fahadmateen@dongguk.edu (F.M.); lnc9428@naver.com (N.L.); 2 Department of Energy and Material Engineering, Dongguk University, Seoul 04620, Korea; asifshzd8@dongguk.edu; 3 Department of Physics, Dongguk University, Seoul 04620, Korea; tajuddins.phy@gmail.com (S.T.U.D.); wyang@dongguk.edu (W.Y.); 4 Research Center for Photoenergy Harvesting & Conversion Technology (phct), Department of Energy and Material Engineering, Dongguk University, Seoul 04620, Korea; ashoksjc88@gmail.com (A.K.K.); jjlee@dongguk.edu (J.-J.L.)

**Keywords:** luminescent solar concentrator, polymer matrix, organic fluorophore, intramolecular charge transfer, light harvesting

## Abstract

Luminescent solar concentrators (LSCs) provide a transformative approach to integrating photovoltaics into a built environment. In this paper, we report thin-film LSCs composed of intramolecular charge transfer fluorophore (DACT-II) and discuss the effect of two polymers, polymethyl methacrylate (PMMA), and poly (benzyl methacrylate) (PBzMA) on the performance of large-area LSCs. As observed experimentally, DACT-II with the charge-donating diphenylaminocarbazole and charge-accepting triphenyltriazine moieties shows a large Stokes shift and limited re-absorption losses in both polymers. Our results show that thin-film LSC (10 × 10 × 0.3 cm^3^) with optimized concentration (0.9 wt%) of DACT-II in PBzMA gives better performance than that in the PMMA matrix. In particular, optical conversion efficiency (*η*_opt_) and power-conversion efficiency (*η*_PCE_) of DACT-II/PBzMA LSC are 2.32% and 0.33%, respectively, almost 1.2 times higher than for DACT-II/PMMA LSC.

## 1. Introduction

**Figure 1:**
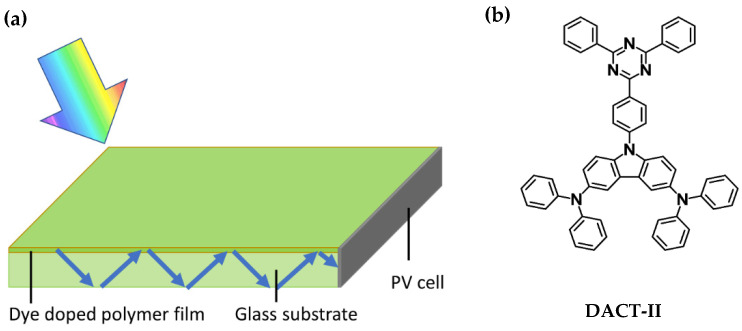
(**a**) Schematic representation of thin-film LSC. (**b**) Chemical structure of DACT-II employed in our study.

Due to rapid urbanization, a considerable increase in global energy consumption has been observed over the past several decades. Currently, buildings utilize around 30% of energy worldwide, due to cooling, heating, and artificial-lighting loads [1,2]. To meet this huge energy demand, substantial attention has been paid to clean and renewable energy technologies, especially grid-free building-integrated photovoltaics (BIPVs) [3]. Among many BIPVs, luminescent solar concentrators (LSCs) offer a cost-effective solution to harness solar energy, while warranting their compatibility with the existing and new infrastructures [4]. Typically, LSCs are fabricated in two simple architectures, namely bulk and thin-film LSC. In the case of bulk LSC, light-emissive fluorophores are embedded in the optically transparent slab of polymer, while a thin-film LSC consists of fluorophores mixed with the polymer matrix to form a thin film on the haze-free glass. In both cases, fluorophores absorb incident sunlight and re-emit it at longer wavelengths. The re-emitted photons are trapped within the polymer slab or glass substrate due to the total internal reflection (TIR) process and are directed to its edges, where they are transformed into electricity by attached PV cells (Figure 1) [5,6]. Recently, it has been suggested that LSCs’ application is not just limited to BIPVs, but they can be applied to various other platforms, such as greenhouses [7], noise barriers [8], indoor decorative elements [9,10], medical devices [7,11], indoor light-harvesting glass [12], and sunroofs of vehicles [13].

Dating back to the 1970s, LSCs were first introduced as an inexpensive alternative to traditional photovoltaics [14]. However, the recent LSCs still offer stability issues [15,16] and reduced efficiency mainly due to re-absorption losses that occur due to the small Stokes shift of fluorophores [17,18], limited fluorophore–polymer compatibility [19], and low photoluminescent quantum yield (PLQY) of fluorophores [20]. Major research efforts devoted to LSCs include (1) the use of suitable fluorophores, such as organic dyes, e.g., coumarins [21], perylenes [22], aggregation induce emissive molecules [23,24,25], π-conjugated polymers [26,27], rare earth complexes [28], and semiconducting quantum dots QDs (core/shell, carbon, and silicon) [29,30,31,32]; (2) different designs, e.g., plasmonic LSCs [33,34], fibers structures [35,36], and multi-layer LSCs [36,37,38]; and (3) identifying the appropriate host polymer. The most reported polymer for LSCs is polymethyl methacrylate (PMMA), while other examples include crosslinked fluoro-polymers [39], polycyclic hexyl methacrylate [40], polysiloxanes [41], L-poly(lactic acid) [42], fluorescent proteins [43], cellulose crystals [44], and unsaturated polyesters [45].

In recent work by our group [46,47], bulk PMMA LSCs were fabricated by utilizing thermally activated delayed fluorescence (TADF) dye, 1,2,3,5-tetrakis(carbazol-9-yl)-4,6-dicyanobenzene (4CzIPN) [48]. Moreover, 4CzIPN shows the intramolecular charge transfer (ICT) features between the carbazole and dicyanobenzene moieties that lead to a drastic increase in the Stokes shift. Reduced re-absorption losses, high photostability due to strong steric hindrance, and high PLQY of 4CzIPN make it an excellent candidate for the large-area LSCs.

In this study, we investigated the effect of the host polymer matrix on the performance of LSC incorporating intramolecular charge transfer fluorophore. We fabricated large-area thin-film LSCs (10 × 10 × 0.3 cm^3^) based on another TADF dye, 9-[4-(4,6-diphenyl-1,3,5-triazin-2-yl)phenyl]-N,N,N′,N′-tetraphenyl-9H-carbazole-3,6-diamine, denoted as DACT-II (Figure 1b) [49]. DACT-II consists of electron donor diphenylaminocarbazole and electron-acceptor triphenyltriazine moieties and exhibits ICT characteristics. In particular, PMMA and poly (benzyl methacrylate) (PBzMA) were investigated as host polymer matrices for DACT-II-based thin-film LSCs. Besides synthesis of DACT-II, we report the optical properties and photovoltaic performance of DACT-II-based thin-film LSCs employing PMMA and PBzMA matrices. Our results suggest that the DACT-II-based thin-film LSC with the PBzMA matrix shows an optical efficiency of 2.32%, which is 1.2 times higher than that with the PMMA matrix.

## 2. Materials and Methods

### 2.1. Materials

For the synthesis of DACT-II, all reagents were acquired from Tokyo Chemical Industry (TCI) and Sigma-Aldrich. For the fabrication of LSCs, PMMA and PBzMA were purchased from Sigma-Aldrich.

### 2.2. Synthesis

#### 2.2.1. Synthesis of 3,6-dibromo-9-(4-(4,6-diphenyl-1,3,5-triazin-2-yl)phenyl)-9H-carbazole (1)

First, 2-(4-bromophenyl)-4,6-diphenyl-1,3,5-triazine (0.4 g, 1.0 mmol), 3,6-dibromo-9H-carbazole (0.33 g, 1.0 mmol), bis(tri-tert-butylphosphine)palladium(0) (0.026 g, 0.05 mmol) and sodium tert-butoxide (0.25 g, 2.6 mmol) were dissolved in anhydrous toluene (13 mL) under a nitrogen atmosphere. The mixture was refluxed for 4 h. After cooling down to room temperature, the solution was poured into chloroform and distilled water for extraction. The chloroform layer was washed with distilled water several times and dried over magnesium sulfate. The crude product was filtered by using Celite 545 and purified via column chromatography on silica gel (eluent:dichloromethane/hexane, 1:4, *v*/*v*). The product was dried in a vacuum oven to give a white powder (yield = 0.10 g, 10%). ^1^ H NMR (500 MHz, CDCl_3_): δ 9.04 (d, J = 8.5 Hz, 2 H), 8.83 (d, J = 6.5 Hz, 4 H), 8.24 (s, J = 2.0 Hz, 2 H), 7.76 (d, J = 8.5 Hz, 2 H), 7.67–7.55 (m, 6 H), 7.57 (dd, J = 9.0 Hz, 2.0 Hz, 2 H), 7.41 (d, J = 9.0 Hz, 2 H).

#### 2.2.2. Synthesis of DACT-II

First, (0.10 g, 0.16 mmol), diphenylamine (0.06 g, 0.35 mmol), tris(dibenzylideneacetone)dipalladium(0)-chloroform adduct (0.004 g, 0.004 mmol), 2-dicyclohexylphosphino-2′,4′,6′-triisopropyl-biphenyl (0.01 g, 0.016 mmol) and sodium *tert*-butoxide (0.037 g, 0.384 mmol) were dissolved in anhydrous toluene (5 mL) under a nitrogen atmosphere. The mixture was refluxed for 12 h. After cooling down to room temperature, the solution was poured into chloroform and distilled water for extraction. The chloroform layer was washed with distilled water several times and dried over magnesium sulfate. The crude product was filtered by using Celite 545 and purified via column chromatography on silica gel (eluent:dichloromethane/hexane, 1:2.5, *v*/*v*). The product was dried in a vacuum oven to give a yellow powder (yield = 0.09 g, 69%). ^1^ H NMR (500 MHz, DMSO-d_6_): *δ* 9.03 (d, J = 8.5 Hz, 2 H), 8.80 (d, J = 7.0 Hz, 4 H), 8.05 (s, J = 2.5 Hz, 2 H), 8.00 (d, J = 8.5 Hz, 2 H), 7.76 (t, J = 7.0 Hz, 2 H), 7.71 (t, J = 7.5 Hz, 4 H), 7.60 (d, J = 9.0 Hz, 2 H), 7.27 (t, J = 8.5 Hz, 10 H), 7.00 (d, J = 7.5 Hz, 8 H), 6.96 (t, J = 7.5 Hz, 4 H).

### 2.3. Fabrication of Thin-Film LSCs

To fabricate DACT-II-based thin-film LSCs with PMMA matrix, 10 wt% solutions of PMMA in chloroform were prepared. The solution was then blended with various concentrations of as-synthesized DACT-II (0.1–1.3 wt%). After proper mixing of final solutions, the doctor-blade coating technique was used to make thin film (~60 µm film thickness) on transparent glass substrates of different sizes. Chloroform was slowly evaporated by keeping the sample under room conditions. The same procedure was applied to fabricate DACT-II-based thin-film LSCs with PBzMA matrix.

### 2.4. Measurements

UV–visible spectrophotometer (Perkin Elmer Lambda 35) and fluorescence spectrophotometer (JASCO, FP-8600) were used to obtain absorbance and emission of the samples. To obtain the spectra of edge emitted photons, an integrating sphere connected to a spectrometer (Avantes, ULS2048) was employed. For PV measurements, crystalline silicon (c-Si) PV cells were purchased locally. Highly transparent adhesive (United Adhesives, OE 1582) was used to attach the PV cells with all edges of the fabricated LSCs (10 × 10 × 0.3 cm^3^). Current–voltage measurements were obtained by illuminating the surface of LSCs with a solar simulator (Mc-Science) having a Xenon arc lamp of 160 W equipped with filters to approximate AM 1.5 G spectrum. The irradiance of the illumination source was calibrated before and found to be 100 mW cm^−2^.

## 3. Results and Discussion

**Scheme 1:**
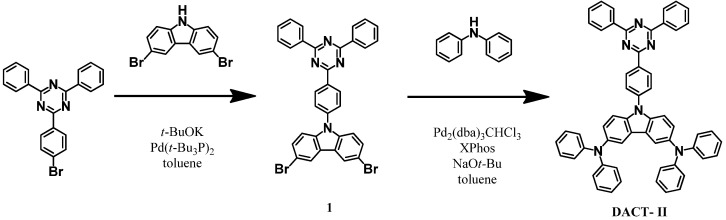
Synthesis of DACT-II.

The synthesis of DACT-II was performed by using a two-step approach (Scheme 1). In detail, Compound 1 was synthesized by 3,6-dibromo-9H-carbazole, bis(tri-tert-butylphosphine) palladium(0) and sodium tert-butoxide under inert environment. Then, diphenylamine, tris(dibenzylideneacetone)dipalladium (0)-chloroform adduct, 2-dicyclohexylphosphino-2′,4′,6′-triisopropyl-biphenyl, and sodium tert-butoxide were reacted in anhydrous toluene under a nitrogen atmosphere, delivering DACT-II in 69% yield after crystallization. The obtained DACT-II was consisted of chemically bonded diphenylaminocarbazole (charge donor) and triphenyltriazine (charge acceptor) moieties.

**Figure 2:**
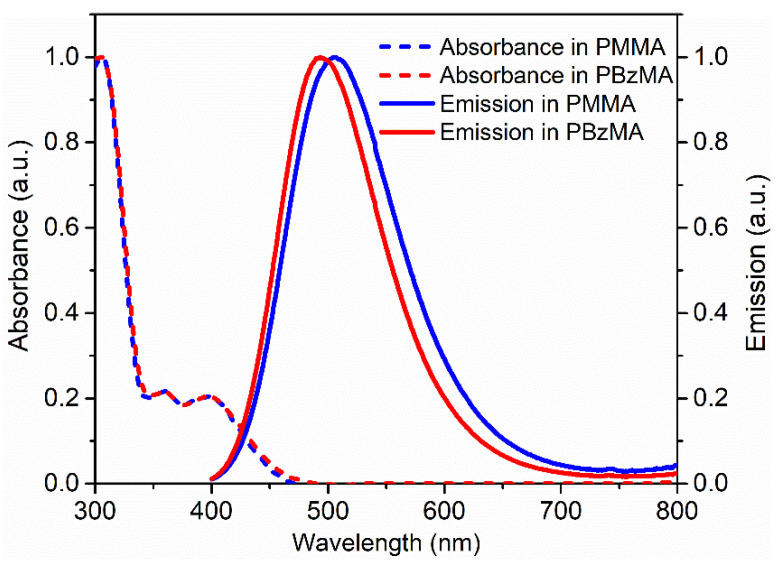
Normalized absorbance and emission spectra of DACT-II in PMMA and PBzMA.

The optical properties of synthesized DACT-II were investigated in PMMA and PBzMA matrices. PBzMA is highly transparent and amorphous that makes it an excellent alternative to the commonly employed PMMA matrix in LSCs. Normalized absorbance and emission spectra of DACT-II in PMMA and PBzMA are displayed in Figure 2. The absorbance range covered the entire ultraviolet (UV) and near UV region, i.e., from 300 to 450 nm. As observed, absorbance is low in the 350–450 nm range, however, this issue can be solved by using a higher concentration of DACT-II in thin-film LSCs. As evident from Figure 2, the absorbance of DACT-II was nearly the same in both polymer matrices. DACT-II exhibited a broad emission with the peak values at 490 and 507 nm in PBzMA and PMMA films, respectively. The expected blue shift in the case of PBzMA is due to the modest polarity of the lateral benzyl group compared to the methyl ester substitution in PMMA. Stokes shift is an important factor in designing an efficient LSC device. Figure 2 also confirms that DACT-II exhibited a large Stokes shift, i.e., less overlap between absorbance and emission spectra in both polymers. Such a large Stokes shift limits the re-absorption losses, even at higher concentrations of DACT-II; thus, it helps improve the LSC efficiency.

**Figure 3:**
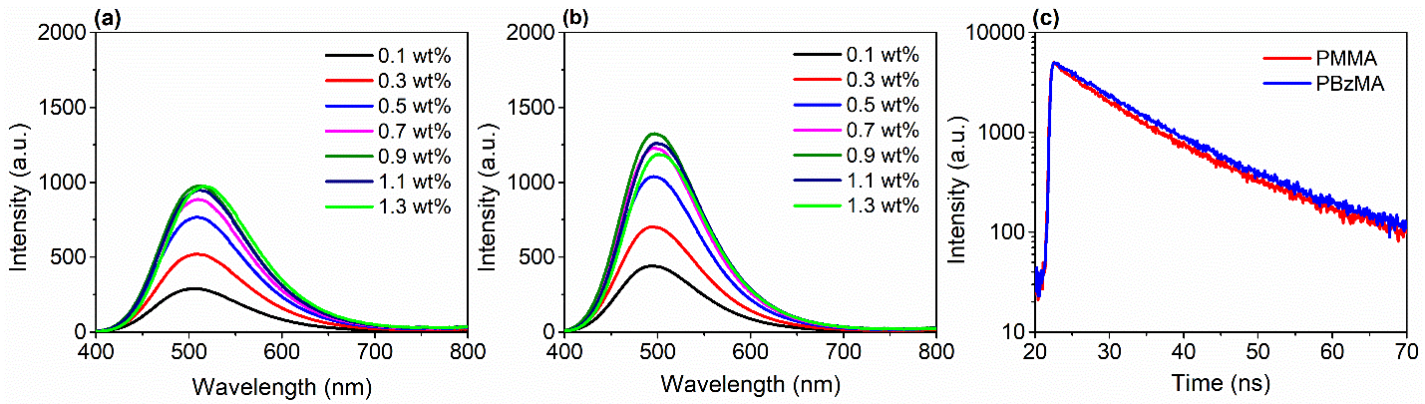
Emission spectra of DACT-II in (**a**) PMMA and (**b**) PBzMA films at the excitation wavelength of 350 nm. (**c**) Time-resolved photoluminescence spectra of DACT-II in PMMA and PBzMA films.

To obtain the optimum concentration of DACT-II in PMMA and PBzMA, thin-film LSCs (5 × 2.5 × 0.1 cm^3^) employing different concentrations, ranging from 0.1 to 1.3 wt%, were fabricated as explained in the experimental section. In Figure 3a, the effect of DACT-II concentration in PMMA film on the emission intensity is reported. For 0.1–0.9 wt% DACT-II loading, a gradual increase in the emission intensity was detected at the excitation wavelength of 350 nm. However, a further rise in the concentration caused a decrease in emission which could be associated with the formation of the aggregate. These aggregates offer sites for non-radiative relaxations of excited state electrons, leading to emission reduction [50]. The limited solubility of DCAT-II in the PMMA matrix is another factor that leads to lowered emission at higher concentrations of DACT-II. Moreover, these spectra were also characterized by a negligible red shift, suggesting modest re-absorption losses. Identical to what was observed for DACT-II/PMMA films, emission intensity (for DACT-II/PBzMA films) improved linearly with DACT-II concentrations up to 0.9 wt% (Figure 3b). Beyond 0.9 wt%, a decrease in the DACT-II emission, along with the progressive red shift, was observed. The comparison of DACT-II in both polymer matrices shows that emission intensity remained higher in PBzMA than PMMA for all the concentrations, making PBzMA a superior alternative to the commonly used PMMA matrix. Our experimental investigations can be justified by the fact that the reduced polarity of PBzMA helps create not only a better dispersion of the DACT-II but also improves the radiative decay channels. To deeply understand the emission mechanism of the DACT-II in PMMA and PBzMA matrices, time-resolved photoluminescent measurements were performed. A single-exponential decay model was employed to fit the photoluminescence decay curve and calculate the excited-state lifetimes. The emission of DACT-II in PMMA at 507 nm decayed with an average excited-state lifetime of 8.6 ns. On the other hand, the excited state lifetime of DACT-II in PBzMA at 490 nm was 9.6 ns (Figure 3c). The decreased excited-state lifetime in the case of the PMMA matrix indicates the possible formation of alternative non-radiative decay channels.

**Figure 4:**
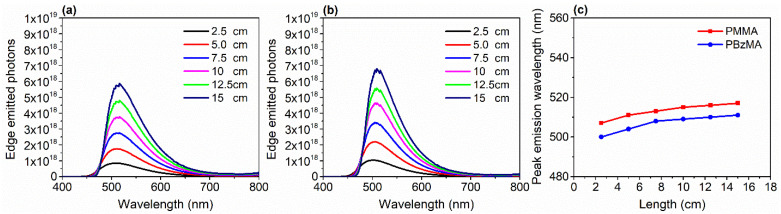
(**a**) Edge emitted photons spectra of DACT-II/PMMA-film LSCs at various lengths. (**b**) Edge emitted photons spectra of DACT-II/PBzMA-film LSCs at various lengths. (**c**) Peak emission wavelengths of LSCs with different lengths. All the LSCs were square-shaped so that length = width.

To investigate the effect of LSC size on the edge emission, we prepared the square-dimensioned thin-film LSCs (DCAT-II concentration 0.9 wt%) with different lengths, and edge emitted photons were obtained by using the integrating sphere method. Figure 4a shows the edge emitted photons spectra of different sized DACT-II/PMMA-film LSCs. The number of edges emitted photons increased linearly with the lengths, which is obvious because, when the size of LSC increases, the total number of incident photons will increase. The same trend was observed in the case of DACT-II based LSC with PBzMA matrix (Figure 4b). Notably, for all the lengths, the total number of photons emitted by the edges of DACT-II-based LSC with PBzMA matrix remained higher than that of the device with PMMA matrix. This trend is consistent with the front-facing emission measurements (Figure 3a,b). To our surprise, a red shift was observed when the size of the LSCs was increased from 2.5 to 15 cm. Peak wavelengths of the edge emission spectra are also presented in Figure 4c. For 2.5 cm length, the peak emission wavelength was 509 and 498 nm for LSCs with PMMA and PBzMA matrices, respectively. Meanwhile, the values changed to 517 and 507 nm for 15 cm–long respective devices. Generally, increment in the size of LSC is accompanied by the escape cone losses, reabsorption losses, and red-shifted edge emissions. The same phenomena have been also noted for LSCs with various designs and using other fluorophores [51,52].

**Figure 5:**
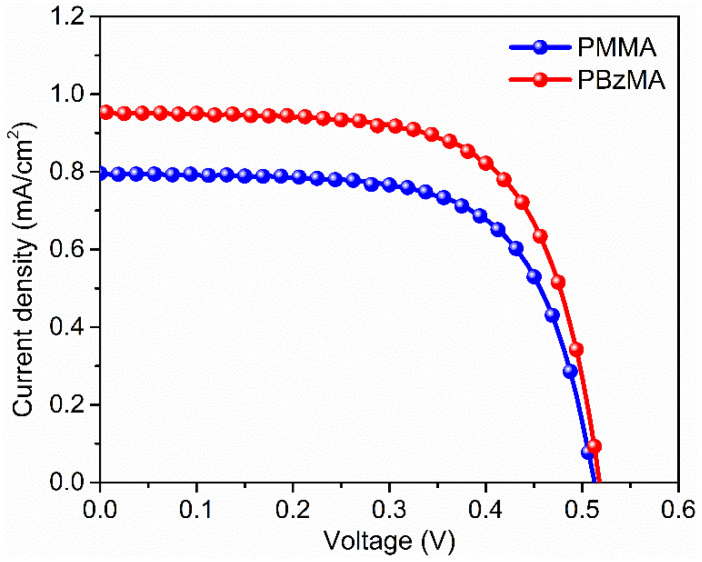
J–V curves of DACT-II based LSC with different polymer matrices.

The potential of DACT-II-based thin-film LSCs as power-producing windows was determined by obtaining optical-conversion efficiency (*η*_opt_) and power-conversion efficiency (*η*_PCE_) of the large-area LSCs (dimension: 10 × 10 × 0.3 cm^3^) having 0.9 wt% of DACT-II in PMMA and PBzMA matrices. Moreover, *η*_opt_ is described as the ratio of LSC edge emitted photons to the total incident photons, while *η*_PCE_ is the ratio of the electrical output to the solar power input. The formula of *η*_opt_ and *η*_PCE_ is given in Equations (1) and (2), respectively.

(1) $$\eta_{\mathrm{opt}\;}=\frac{I_{\mathrm{LSC}}\times {\;A}_{\mathrm{Edges}}\;}{I_{\mathrm{PV}\;\mathrm{cell}}\times {\;A}_{\mathrm{LSC}}}$$

(2) $$\eta_{\mathrm{PCE}}=\frac{\;I_{\mathrm{LSC}}\times V_{\mathrm{OC}}\times FF}{{\;A}_{\mathrm{LSC}}\times {\;F}_{\mathrm{IN}}}$$

where *I*_LSC_ (mA) and *I*_PV cell_ (mA) are short-circuit current obtained by LSC connected PV cell and short-circuit current of bare PV cell(without LSCs attached). A_Edges_ (cm^2^) and A_LSC_ (cm^2^) are the area of LSC edges where PV cells are attached and surface area of LSC. While in Equation (2), *V*_OC_ (V), *FF*, *F*_IN_ (mWcm^−2^) are the open-circuit voltage, fill factor, and the incident solar power density, respectively. It is important to note that PV cell was connected to only one edge while other three edges were masked, and overall LSC was then corrected by multi-plying the current density by 4. Current density–voltage (J–V) curves taken by the DACT-II-based LSC with PMMA and PBzMA matrices are depicted in Figure 5, while the values of other PV parameters and values of *η*_opt_ and *η*_PCE_ are listed in Table 1. It is evident from the results that DACT-II-based LSC with PBzMA matrix outperformed and gave the *η*_opt_ and *η*_PCE_ of 2.32 and 0.33%, respectively. These values are 1.2 times higher than the LSC with the PMMA matrix.

Additionally, an analytical model (Equation (3)) [53] was used to estimate the optical efficiency (*η*_opt_) of large-area LSCs (up to 10,000 cm^2^, for Length = 100 cm) utilizing DACT-II in PMMA and PBzMA matrices.

(3) $$\eta_{\mathrm{opt}}=\left(1-R\right)\;\eta_{\mathrm{abs}}\;.\;\;\eta_{\mathrm{int}}$$

where *R* denotes the reflection losses, which are approximately 4% in the case of polymers with a refractive index of 1.5. Note that PMMA and PBzMA show same refractive index. Moreover, *η*_abs_ and *η*_int_ are the absorption efficiency (Equation (4)) and internal quantum efficiency (Equation (5)), respectively.

(4) $$\eta_{\mathrm{abs}\;}=\frac{\int_{280}^{1100}P_{\mathrm{in}}\left(\lambda \right)\;\left[1-e^{-\alpha \left(\lambda \right)t}\right]d\lambda }{\int_{280}^{1100}P_{\mathrm{in}}\left(\lambda \right)d\lambda }$$

(5) $$\eta_{\mathrm{int}\;}=\frac{\int_{0}^{\infty }\frac{\eta_{\mathrm{QY}\;}\eta_{\mathrm{trap}\;}}{1+\beta \alpha \left(\lambda \right)\frac{t\;}{D}L\left(1-\eta_{\mathrm{QY}\;}\eta_{\mathrm{trap}\;}\right)}\;I_{\mathrm{PL}}\left(\lambda \right)d\lambda }{\int_{0}^{\infty }I_{\mathrm{PL}}\left(\lambda \right)d\lambda }$$

**Figure 6:**
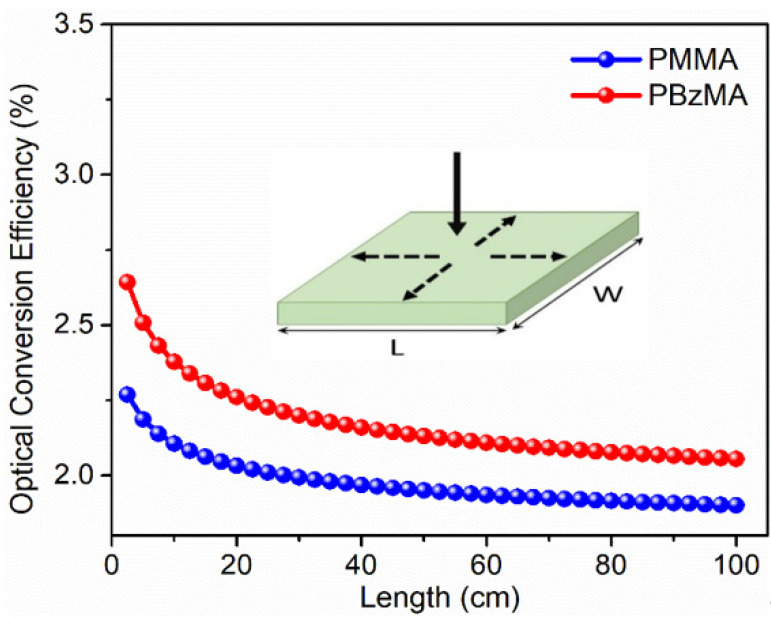
Calculated optical conversion efficiency of DACT-II-based LSCs with PMMA and PBzMA matrices. For all *η*_opt_ values, the LSCs were assumed to be in square shape (length = width).

In Equation (4), *α* is the absorption coefficient of DACT-II in polymeric films, t is the thickness of the film and *P*_in_ is the incident photon flux. In Equation (5), *η*_QY_ is the PLQY of DACT-II (49 and 56% in PMMA and PBzMA, respectively); *η*_trap_ is a light-trapping efficiency, which is around 75% for a given system; *β* is a numerical factor and is equal to 1.4 [37]; *I*_PL_ is an emission intensity; and D and L represent the thickness and length of a whole LSC device. Moreover, LSCs are assumed to be square such that the length of the LSC is equal to the width. As shown in Figure 6, *η*_opt_ drops with the increasing length of LSCs with PMMA and PBzMA matrices. In the case of DACT-II-based LSC with PMMA matrix, the calculated *η*_opt_ was 2.25 and 1.85% for the length 2.5 and 100 cm, respectively. When the PBzMA matrix was employed, *η*_opt_ soared to 2.64% for 2.5 cm and 2.05% for the 100 cm long LSCs. The calculated *η*_opt_ was based on the emissions from all edges of the LSC device as shown in the insert of Figure 6. A drop in *η*_opt_ is expected, since the re-emitted photons are more susceptible to optical losses at higher lengths of LSCs, as can be seen in other studies [25,29]. Although the overall *η*_opt_ of our fabricated LSCs is low, which is due to the low absorption range (300–450 nm), our results confirm that PBzMA can be applied as a potential alternative to commonly employed PMMA matrix for most of the organic fluorophores based LSCs.

## 4. Conclusions

In summary, we demonstrated the effect of polymer matrices on the DACT-II-based LSCs. First, we synthesized the DACT-II, a TADF dye with intramolecular charge transfer characteristics, and blended it with PMMA and PBzMA to make large-area thin-film LSCs. At the optimized concentration (0.9 wt%), DACT-II-based LSC with PBzMA matrix showed 2.32 and 0.33% of *η*_opt_ and *η_PCE_*, respectively. Conversely, the *η*_opt_ and *η_PCE_* of the device with PMMA matrix were 1.92 and 0.28%, respectively. Better efficiencies in the case of PBzMA are attributed to more efficient dispersion of the DACT-II in PBzMA, which makes PBzMA a better choice for the fabrication of thin-film LSCs.

## Figures and Tables

**Table 1:** Photovoltaic parameters of DACT-II-based LSC (10 × 10 × 0.3 cm^3^) with different polymer matrices.

Samples	*V*oc (V)	*I*sc (mA)	*FF* (%)	*η*_opt_ (%)	*η*_PCE_ (%)
DCAT-II/PMMA-based LSC	0.51	79.44	66.57	1.92	0.28
DCAT-II/PBzMA-based LSC	0.51	97.10	66.70	2.32	0.33

## Data Availability

Not applicable.
